# Cancer Risk in Patients with Down Syndrome—A Retrospective Cohort Study from Germany

**DOI:** 10.3390/cancers16061103

**Published:** 2024-03-09

**Authors:** Sarah Krieg, Andreas Krieg, Sven H. Loosen, Christoph Roderburg, Karel Kostev

**Affiliations:** 1 Department of Inclusive Medicine, University Hospital Ostwestfalen-Lippe, Bielefeld University, 33617 Bielefeld, Germany; 2 Department of General and Visceral Surgery, Thoracic Surgery and Proctology, University Hospital Herford, Medical Campus OWL, Ruhr University Bochum, 32049 Herford, Germany; andreas.krieg@klinikum-herford.de; 3 Department of Gastroenterology, Hepatology and Infectious Diseases, University Hospital Duesseldorf, Medical Faculty, Heinrich Heine University Duesseldorf, 40225 Duesseldorf, Germany; sven.loosen@med.uni-duesseldorf.de (S.H.L.); christoph.roderburg@med.uni-duesseldorf.de (C.R.); 4 Epidemiology, IQVIA, 60549 Frankfurt, Germany; karel.kostev@iqvia.com

**Keywords:** Down syndrome, cancer, cancer risk, intellectual disorder, breast cancer, prevention

## Abstract

**Simple Summary:**

Individuals with Down syndrome (DS) are thought to have a unique tumor profile. This study retrospectively compared patients with DS aged ≥18 years from primary care practices with patients without DS between 2005 and 2021 for cancer incidence after matching for age, sex, average annual visit frequency, and comorbidity. The 5-year cumulative incidence of cancer overall and specific cancers was analyzed using Kaplan–Meier curves and compared using the log-rank test. In addition, univariable Cox regression analysis was performed. A total of 2438 patients with DS and 12,190 patients without DS were included. Regression analysis showed no significant association between DS and subsequent cancer in the overall population. Analyses by cancer type and sex showed a strong but non-significant negative association between DS and breast cancer in women. Our results may provide the basis for future studies to clarify whether and to what extent an adapted screening program needs to be modified for individuals with Down syndrome due to the particular cancer distribution pattern.

**Abstract:**

Background: Individuals with Down syndrome are thought to have a unique tumor profile. Methods: Using the IQVIA Disease Analyzer database, patients aged ≥18 years diagnosed with Down syndrome in German general practices between 2005 and 2021 were compared with patients without Down syndrome for cancer incidence, adjusting for age, sex, average annual visit frequency, and comorbidity. The 5-year cumulative incidence of cancer overall and specific cancers was analyzed using Kaplan–Meier curves and compared using the log-rank test. In addition, univariable Cox regression analysis was performed. Results: A total of 2438 patients with Down syndrome and 12,190 patients without Down syndrome were included; 3.9% of patients without Down syndrome and 3.1% of patients with Down syndrome were diagnosed with cancer (*p* = 0.143). Regression analysis showed no significant association between Down syndrome and subsequent cancer in the total population (HR: 0.79; 95% CI: 0.57–1.09), in women (HR: 0.89; 95% CI: 0.56–1.37), or in men (HR: 0.69; 95% CI: 0.43–1.11). Analyses by cancer type and sex showed a strong but not significant negative association between Down syndrome and breast cancer in women (HR: 0.33; 95% CI: 0.12–0.93). Conclusions: Our results could form the basis for future studies to clarify whether and to what extent an adapted screening program needs to be modified for individuals with Down syndrome due to the particular cancer distribution pattern.

## 1. Introduction

Down syndrome (DS) is the most common chromosomal abnormality in humans with an incidence of approximately 1:800 live births and a range of characteristic phenotypic features and medical problems [1,2,3]. While it is well established that DS is associated with a significantly increased risk of leukemia in childhood [4,5], less is known about the development of cancer in adulthood among individuals with DS, with data on the incidence and distribution of solid tumors often conflicting [6,7]. However, given the increased life expectancy of individuals with DS in recent decades, mainly due to the improved treatment of congenital heart defects, the risk of cancer in adulthood is becoming increasingly important [8]. Studies suggest a lower incidence of solid tumors in individuals with DS, but results have been limited by the relatively small proportion of older individuals included [4,9,10,11]. In this context, three epidemiological studies on cancer incidence have shown that individuals with DS have a twofold lower risk of developing solid tumors than the general population [6,7,10,11]. In addition, a US mortality study including approximately 18,000 patients with DS showed that a person with DS is 50 to 100 times less likely to die from a form of tissue cancer than the general population when compared with individuals of the same age and sex [9]. 

Finally, there is no consensus on recommendations regarding cancer treatment and follow-up for individuals with DS [6]. Additional knowledge about cancer risk in individuals with DS could help to modify screening strategies in this specific population and to develop new approaches for cancer prevention and treatment in the general population. Therefore, the aim of this study was to investigate cancer incidence in a large, real-life cohort of individuals with DS within the German general population.

## 2. Material and Methods

### 2.1. Database

This retrospective cohort study was based on data from the Disease Analyzer database (IQVIA), which contains drug prescriptions, diagnoses, and basic medical and demographic data obtained directly and anonymously from the computer systems used in the practices of general practitioners and specialists [12]. The database covers approximately 3% of all private practices in Germany. The sampling method for the Disease Analyzer database is based on summary statistics from of all physicians in Germany published annually by the German Medical Association. IQVIA uses these statistics to determine the panel design based on four strata including specialty, state, community size category, and physician age. The panel of practices included in the Disease Analyzer database has previously been shown to be representative of general and specialist practices in Germany [12]. Finally, this database has already been used in a number of studies focusing on cancer [13,14].

### 2.2. Study Population

**Figure 1:**
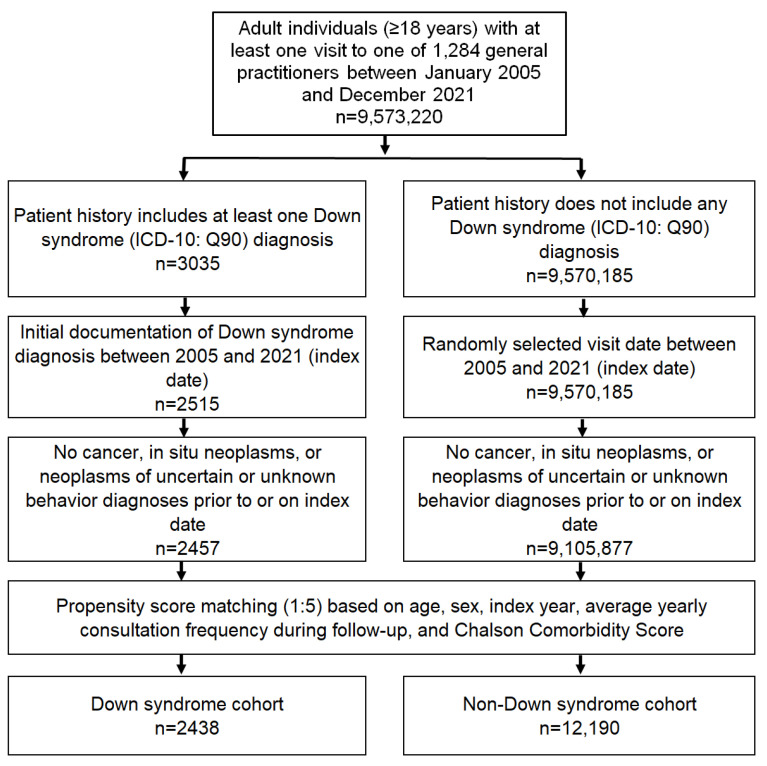
Selection of study patients.

This study included adult patients (≥18 years) with an initial diagnosis of Down syndrome (ICD-10: Q90) in 1284 general practices in Germany between January 2005 and December 2021 (index date; Figure 1). Patients with a diagnosis of cancer (ICD-10: C00–C97), in situ neoplasia (ICD-10: D00–D09), and neoplasia of uncertain or unknown behavior (ICD-10: D37–D48) before, on or within three months of the index date were excluded.

After applying similar inclusion criteria, individuals without DS were matched to DS patients using nearest-neighbor propensity score matching (1:5) based on age, sex, index year, average annual visit frequency during follow-up, and Charlson Comorbidity Score (excluding cancer diagnoses) [15]. The Charlson Index is a weighted index that accounts for the number and severity of comorbidities in administrative database studies, and includes a wide range of comorbidities (macrovascular diseases, pulmonary diseases, gastrointestinal, liver, and renal diseases, diabetes, AIDS, and others) [15]. For the non-DS cohort, the index date was that of a randomly selected visit between January 2005 and December 2021 (Figure 1).

### 2.3. Study Outcomes

The study outcomes were first diagnoses of cancer overall (ICD-10: C00–C97) and first diagnoses of cancer of the esophagus and stomach (ICD-10: C15, C16), colon/rectum (ICD-10: C18, C20), pancreas (ICD-10: C25), lung (ICD-10: C34), skin (ICD-10: C43), female breast (ICD-10: C50), female genital organs (ICD-10: C51–C59), prostate (ICD-10: C61) and testis (ICD-10: C62), as well as lymphoma (ICD-10: C81–C88) and leukemia (ICD-10: C91–C95), up to five years after the index date as a function of DS.

### 2.4. Statistical Analyses

Differences in the sample characteristics and diagnosis prevalence between the DS and non-DS cohorts were compared using the Wilcoxon signed-rank test for continuous variables, the McNemar test for categorical variables with two categories, and the Stuart–Maxwell test for categorical variables with more than two categories.

The 5-year cumulative incidence of cancer overall and for defined cancer types was also studied using Kaplan–Meier curves, and these curves were compared using the log-rank test. Finally, a univariable Cox regression analysis was conducted to examine the association between DS and cancers. These models were applied separately for female and male individuals. The results of the Cox regression model are displayed as hazard ratios (HRs) and 95% confidence intervals (CIs). *p*-values of <0.01 were considered statistically significant due to the multiple comparisons performed. Analyses were conducted using SAS version 9.4 (SAS Institute, Cary, NC, USA).

## 3. Results

### 3.1. Baseline Characteristics of the Study Cohort

The present study included 2438 individuals with DS and 12,190 individuals without DS. The baseline characteristics of the study cohort are shown in Table 1. The mean age was 41.8 (SD: 15.3) years, and 48.0% of patients were female. Patients had an average of 5.2 visits per year during the follow-up period.

### 3.2. Cumulative Incidence of Cancer among Patients with and without DS

**Figure 2:**
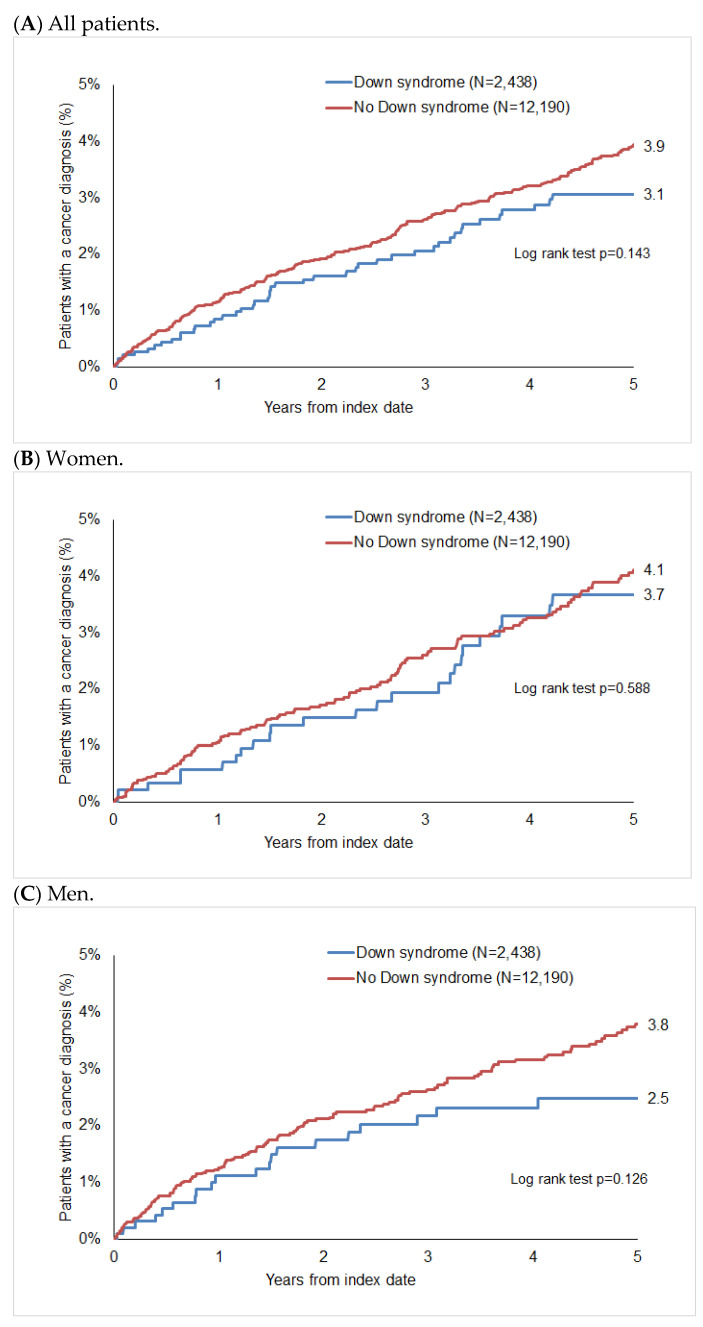
Cumulative incidence of cancer in patients with and without Down syndrome.

After up to five years of follow-up, 3.9% of non-DS patients and 3.1% of DS patients were diagnosed with cancer (*p* = 0.143, Figure 2), with 4.1% of women without DS and 3.7% of women with DS, and 3.8% vs. 2.5% of men without and with DS, respectively, being diagnosed with cancer during this period. Although we found that fewer DS patients were diagnosed with cancer in the total population and in the group of men (Figure 2A,C), these differences were not significant.

### 3.3. Association between Down Syndrome and Cancer Diagnosis

In the regression analysis, we found no significant association between DS and subsequent cancer in the total population (HR: 0.79; 95% CI: 0.57–1.09), in women (HR: 0.89 95% CI: 0.56–1.37), or in men (HR: 0.69; 95% CI: 0.43–1.11). The analyses stratified by cancer type and sex indicated a strong but not significant negative association between DS and breast cancer in women (HR: 0.33; 95% CI: 0.12–0.93, Table 2). In addition, a strong positive but not significant association was observed between DS and female genital tract cancer (HR: 2.54; 95% CI: 0.99–6.56), as well as leukemia (HR: 6.50; 95% CI: 1.08–39.00) and testicular cancer (HR: 1.73; 95% CI: 0.34–8.92) in men (Table 2).

## 4. Discussion

In this large German cohort study based on data from the Disease Analyzer database (IQVIA), 2438 adult patients (≥18 years) with DS were compared with a cohort of patients without DS for cancer incidence. The results show no significant association between DS and cancer risk in the overall population. However, analyses by cancer type and sex show a strong but non-significant negative effect of DS on breast cancer in women, and a positive effect on cancers of the female genital tract and on leukemia and testicular cancer in men.

Overall, the present findings are consistent with the observations of most studies, including age-related epidemiological surveys, which show that solid tumors are generally rather rare in individuals with DS [7,16].

In addition to the present study, Baksh et al. conducted a population-based cohort study using electronic health records from 1990 to 2020 in the United Kingdom, which included 10,204 individuals with DS and 69,150 individuals with intellectual disability (ID). The aim was to identify differences in the disease patterns of individuals with DS across the lifespan, as well as syndrome-specific health conditions and their prevalence as a function of age. Interestingly, they found that individuals with DS show distinct age-related patterns and incidences that differ from the general population, and even from individuals with other IDs [17].

Similarly to our study, Hasle et al. examined a cohort of 3530 individuals with DS for cancer incidence using registry data from Denmark. They found that the overall risk of solid tumors was reduced in individuals with DS, especially in those aged 50 years and older. Interestingly, the incidence of breast cancer in particular was significantly lower in women, which is in line with the results of our study [7]. In fact, breast cancer has been found to be less common in individuals with DS in a number of other epidemiological studies on incidence and mortality in individuals with DS [6,9]. The first report of a lower incidence of breast cancer in women with DS was published more than 40 years ago [18]. Regarding breast cancer screening, some authors suggest the same screening strategies for women with DS aged 50 to 69 or 74 years as for the general population, i.e., biannual mammography every 2 years in accordance with the recommendations for women with ID [19,20].

A review of the literature on cancer screening in adults with DS by Rethoré et al. has considered the tumor profile in this specific population and includes five epidemiological studies on cancer incidence in individuals with DS, four studies on cancer mortality, and five guidelines on medical surveillance in individuals with DS. The authors conclude that solid tumors are at most half as common in adults with DS as in the general population. For breast cancer in women with DS specifically, the authors estimated the incidence to be up to ten times lower than in the general population (in the general population, approximately one in eight women are affected) [6]. Interestingly, a study investigating the benefits of mammography screening in a group of women with DS found only two cases of cancer (0.7%) among 684 patients, one of which was a non-invasive ductal carcinoma in situ and the other a phyllodes tumor with borderline malignant potential that had already been detected by palpation prior to mammography [21]. Another study by Alagoz et al. evaluated the risk–benefit ratio of different mammography screening strategies in 1000 women with DS in the USA. The authors concluded that the benefit–harm ratio in terms of life years gained and harm from false-positive findings in a population with limited life expectancy and low cancer risk makes current breast cancer screening guidelines inappropriate for this specific population [22]. Based on these findings, other studies have also suggested that individuals with DS should be excluded from mass screening programs, and instead have annual clinical follow-ups and ultrasound or MRI in suspected cases [6,19,23,24]. Although breast cancer is less common in people with DS, as shown in our study, it is important to remember that it does occur. Especially in this population, cognitive difficulties and possibly impaired sensory and motor skills may prevent women from recognizing a suspicious breast lesion via self-palpation. Therefore, the appropriateness of less intensive breast screening in individuals with DS needs to be carefully considered. Further studies, especially with a prospective design, are needed to clarify this issue. In general, as noted above, the incidence, spectrum, and profile of tumors in individuals with DS appear to be very specific [6,17,25]. In this context, the risk of cancer in individuals with DS even differs from the risk of cancer in persons with any other disorder associated with ID. Studies have shown that breast cancer, for example, is no less common in the overall group of women with ID than in the general population [26]. This may be surprising as they share the same risk factors as women with DS, namely, overweight and obesity, physical inactivity, and very low pregnancy and breastfeeding rates [27,28,29]. It is also interesting to note that the tumor profile in patients with DS is reported to be different from that in those with trisomy 18 (Edwards syndrome) or trisomy 13 (Patau syndrome) [30,31].

There are conflicting results in the literature regarding our findings of an increased incidence of female genital cancers in women with DS. For example, earlier studies found a non-significant increase in the incidence of ovarian cancer in women with DS [32,33], but a lower mortality from ovarian cancer [9]. However, Hasle et al. concluded that the risk of ovarian cancer in patients with DS was probably comparable to that of the general population [7]. By contrast, they and other authors found a significantly lower risk of cervical cancer in patients with DS [7,33]. In addition, our study shows evidence of an increased incidence of testicular tumors in patients with DS, although the results are not significant. This finding is also consistent with observations in the literature; indeed, several epidemiologic studies report a three- to fivefold increased incidence of testicular tumors in people with DS [7,9,10,32,33,34,35]. However, the underlying mechanism for this potential increased incidence remains unclear. Risk factors such as cryptorchidism and microlithiasis as well as genetic aspects have been discussed [6]. Regarding recommendations for cancer screening, Rethoré et al. suggest in their review that women with DS should be offered cervical cancer screenings from the age of 25, and that men between the ages of 15 and 45 should be offered annual testicular cancer screenings through palpation of the testicles by a specialist [6]. Further studies are needed to better understand the risk and assess the need for tailored screening.

Several underlying pathophysiological mechanisms for the lower risk of solid tumors in patients with DS have been discussed. These include an increased rate of apoptosis in cells with DS, which could result in cell death being a more frequent response to DNA damage [36]. The protective effect against solid tumors has also been attributed to the increased expression of 1 or more of the 231 additional genes on the extra copy of chromosome 21. Baek and colleagues found that the inhibition of tumor development in individuals with DS may be due in part to the suppression of tumor angiogenesis, specifically the increased expression of DS candidate region 1 (Dscr1, RCAN1), which encodes a protein that suppresses vascular endothelial growth factor (VEGF)-mediated angiogenic signaling via the calcineurin pathway [36,37,38,39].

To date, few population-based studies have investigated the association between DS and the incidence of different cancers, and, to the best of our knowledge, this is the first study in Germany to do so. The strength of this study lies in the matching of individuals with DS to the general population in terms of age, sex, comorbidity index, and frequency of visits.

However, we also need to acknowledge some limitations of the study. Many limitations are due to the design of the study and are therefore unavoidable. As the diagnoses are based on coding, we cannot exclude the possibility of miscoding or undercoding. As the numbers for DS were relatively small, we used 1:5 matching for the analysis, and the patients included were not restricted to those who were followed for at least one year from the index date. It should also be noted that despite increasing life expectancy, DS is still often associated with premature mortality, which may have affected the incidence estimates. Some cancers occur later in life, usually up to the age of 60–70 years, which may explain the small number of cancer cases in patients with DS. Finally, some cancers were not common enough for any difference to be discernible. Although our data show a possible trend in some such cases, the results did not reach significance. As mentioned above, another limitation of this study is that it included patients with DS aged 18 years and older. However, it is possible that patients with DS may receive pediatric treatment for longer than 18 years. As a result, diagnoses made in childhood, adolescence or possibly young adulthood may not be included in our analyses. For example, it is well known that acute leukemias in particular have their main incidence in early childhood [7]. This could explain why we did not find a significantly higher incidence of leukemia among patients with DS in our study. However, the question of why we found a positive, although not significant, association between leukemia and Down syndrome only in men and not in women remains unanswered and requires further research. Another limitation of this study is that no information was available on cancer risk factors (e.g., tobacco use and sexual activity), tumor stage, or cancer mortality. Such details would be of interest for further analysis, especially for health services research, as there are studies showing that tumors in patients with mental and physical disabilities are often diagnosed at a late stage when curative treatment is no longer possible [6].

It is also important to note that there are still inequalities in the surveillance, diagnosis, and treatment of health problems in individuals with ID, including individuals with DS, which means that tumors may go undetected. It is also possible that individuals with DS are less likely to report symptoms or to have cancer screening tests such as mammograms. Finally, our study can only show associations, and not prove causation. Further research is needed to build on our findings and find out more about the links between different conditions and DS in individuals with the condition.

## 5. Conclusions

Overall, our study showed no significant differences in cancer incidence between individuals with and without DS. However, a specific distribution of cancers in individuals with DS may have implications for cancer screening programs conducted in the general population. Our findings could provide a basis for future studies to clarify whether and to what extent an adapted screening program for certain cancers needs to be modified for individuals with DS.

## Figures and Tables

**Table 1:** Baseline characteristics of the study sample (after 1:5 propensity score matching).

Variable	Proportion among Patients with Down Syndrome (*N*, %) *N* = 2438	Proportion among Patients without Down Syndrome (*N*, %) *N* = 12,190	*p*-Value
Age (Mean, SD)	41.8 (15.3)	41.8 (15.3)	0.919
Age 18–30	720 (29.5)	3587 (29.4)	0.970
Age 31–40	438 (18.0)	2241 (18.4)	
Age 41–50	490 (20.1)	2432 (20.0)	
Age 51–60	497 (20.4)	2426 (19.9)	
Age >60	293 (12.0)	1504 (12.3)	
Female	1171 (48.0)	5855 (48.0)	1.000
Male	1267 (52.0)	6335 (42.0)	
Number of physician visits per year during the follow-up (Mean, SD)	5.2 (4.8)	5.2 (4.8)	1.000
Charlson Comorbidity Score (CCS)	0.4 (0.9)	0.4 (0.9)	1.000
CCS 0	1806 (74.1)	9030 (74.1)	1.000
CCS 1	383 (15.7)	1915 (15.7)	
CCS 2	153 (6.3)	770 (6.3)	
CCS >2	95 (3.9)	475 (3.9)	
Index year 2005–2008	375 (15.4)	1875 (15.4)	1.000
Index year 2009–2012	570 (23.4)	2850 (23.4)	
Index year 2013–2016	643 (26.4)	3215 (26.4)	
Index year 2017–2021	850 (34.8)	4250 (34.8)	

Proportions of patients given in *N*, % unless otherwise indicated. SD: standard deviation.

**Table 2:** Association between Down syndrome and subsequent cancer diagnosis in patients followed in general practices in Germany (univariable Cox regression models).

Outcome Diagnosis	Incidence (Cases per 1000 Patient Years) among Down Syndrome Patients	Incidence (Cases per 1000 Patient Years) among Non-Down Syndrome Patients	HR (95% CI)	*p*-Value
All patients				
Cancer total	3.89	5.53	0.79 (0.57–1.09)	0.144
Esophagus/stomach	0.09	0.11	0.85 (0.10–7.29)	0.883
Colon/rectum	0.17	0.43	0.99 (0.40–2.41)	0.975
Pancreas	0.26	0.16	1.66 (0.44–6.26)	0.455
Bronchus and lung	0.09	0.50	0.26 (0.06–1.10)	0.067
Skin	0.17	0.23	0.77 (0.22–2.67)	0.680
Lymphoma	0.17	0.25	0.68 (0.15–3.07)	0.619
Leukaemia	0.26	0.09	1.75 (0.61–5.06)	0.298
Women				
Cancer total	4.49	5.65	0.89 (0.58–1.37)	0.588
Esophagus/stomach	0.18	0.14	1.40 (0.15–13.43)	0.773
Colon/rectum	0.36	0.48	0.81 (0.18–3.72)	0.790
Pancreas	0.18	0.14	1.49 (0.15–13.43)	0.730
Bronchus and lung	0	0.34	----- *	
Skin	0.36	0.24	1.68 (0.33–8.68)	0.535
Female breast	0.36	1.30	0.33 (0.12–0.93)	0.036
Female genital organs	0.90	0.43	2.54 (0.99–6.56)	0.054
Lymphoma	0	0.10	----- *	
Leukaemia	0	0.10	----- *	
Men				
Cancer total	3.33	5.43	0.69 (0.43–1.11)	0.128
Esophagus/stomach	0	0.09	----- *	
Colon/rectum	0	0.39	----- *	
Pancreas	0.33	0.17	2.27 (0.42–12.39)	0.345
Bronchus and lung	0.17	0.65	0.30 (0.04–2.25)	0.240
Skin	0	0.22	----- *	
Prostate	0.17	0.82	0.35 (0.08–1.46)	0.149
Testis	0.33	0.22	1.73 (0.34–8.92)	0.514
Lymphoma	0.33	0.39	0.96 (0.21–4.44)	0.958
Leukaemia	0.50	0.09	6.50 (1.08–39.00)	0.041

* Odds ratio cannot be evaluated in at least one cohort, no cancer diagnoses were documented.

## Data Availability

The data that support the findings of this study are available from the corresponding author on reasonable request.
